# The PIT-trap—A “model-free” bootstrap procedure for inference about regression models with discrete, multivariate responses

**DOI:** 10.1371/journal.pone.0181790

**Published:** 2017-07-24

**Authors:** David I. Warton, Loïc Thibaut, Yi Alice Wang

**Affiliations:** 1 School of Mathematics and Statistics and the Evolution & Ecology Research Centre, UNSW Sydney, NSW, Australia; 2 School of Mathematics and Statistics, UNSW Sydney, NSW, Australia; 3 Institute of Computational and Theoretical Studies, Department of Computer Science, Hong Kong Baptist University, Hong Kong SAR, China; University of California, San Diego, UNITED STATES

## Abstract

Bootstrap methods are widely used in statistics, and bootstrapping of residuals can be especially useful in the regression context. However, difficulties are encountered extending residual resampling to regression settings where residuals are not identically distributed (thus not amenable to bootstrapping)—common examples including logistic or Poisson regression and generalizations to handle clustered or multivariate data, such as generalised estimating equations. We propose a bootstrap method based on probability integral transform (PIT-) residuals, which we call the PIT-trap, which assumes data come from some marginal distribution *F* of known parametric form. This method can be understood as a type of “model-free bootstrap”, adapted to the problem of discrete and highly multivariate data. PIT-residuals have the key property that they are (asymptotically) pivotal. The PIT-trap thus inherits the key property, not afforded by any other residual resampling approach, that the marginal distribution of data can be preserved under PIT-trapping. This in turn enables the derivation of some standard bootstrap properties, including second-order correctness of pivotal PIT-trap test statistics. In multivariate data, bootstrapping rows of PIT-residuals affords the property that it preserves correlation in data without the need for it to be modelled, a key point of difference as compared to a parametric bootstrap. The proposed method is illustrated on an example involving multivariate abundance data in ecology, and demonstrated via simulation to have improved properties as compared to competing resampling methods.

## Introduction

The bootstrap is a generally applicable inferential tool that is so intuitive and widely used in statistics that it not only appears in most university courses, but it has also been proposed for teaching in high schools. A key challenge however arises when applying the bootstrap to regression models with a non-Gaussian response. Common examples include logistic and Poisson regression, and extensions to handle clustered or multivariate data such as generalised estimating equations [1]. Specifically, regression models are specified conditionally on the observed explanatory variables, so bootstrapped values should usually be generated conditionally, with the set of design points remaining fixed in resamples. One way to achieve this is to use a parametric bootstrap [2], although this requires specification of a fully parametric model for the data, which can be a challenge in high-dimensional settings. A non-parametric bootstrap that keeps the design fixed can be achieved in models with additive errors, by estimating residuals and then resampling them in some way [3, 4]. But for non-Gaussian regression models, it is sometimes not obvious how residuals should be defined. For example, when bootstrapping generalized linear models, Pearson, deviance or Anscombe residuals have been considered [2] but none of these are identically distributed, even in large samples. Some have proposed resampling quantities in the estimating equations [5–7], but for non-normal responses these are also not identically distributed, even in large samples. We will see later that resampling from non-identical distributions can lead to undesirable properties in resultant resampling procedures.

Table 1 is a well-known example data set from ecology [8] which serves to highlight the problems of current bootstrap methodology. The data are multivariate counts of invertebrates (copepods) collected in a randomized blocks design along beaches in Tasmania, Australia, to study the nature of the effect of crab exclusion on communities of small invertebrates. The design has two treatment replicates and two control replicates in each of four blocks, and we would like to start by testing the hypothesis of no interaction between treatment and block. Notice that the number of variables (*p* = 12) is not small compared to the number of observations (*n* = 16), meaning that we cannot rely on standard large-*n*-fixed-*p* inference. One way to address this issue is to use a resampling approach for inference, where we resample rows of data to make inferences that are robust to misspecification of the correlation between the twelve species [9, 10].

**Table 1:** A 16 × 12 matrix of copepod abundances, classified to species. Data taken from a study at Eagle Neck, Tasmania [8], in which twelve species of copepods (a type of small crustacean) were sampled at 16 transects in each of 4 sites. At each site, crabs had disturbed two transects and left two undisturbed. It is of interest to determine if there is an effect of disturbance and if it interacts with block, collectively across all twelve species.

Treatment	Block	*Am*	*Ad*	*Ec*(a)	*Ec*(b)	*Ha*	*Le*(a)	*Le*(b)	*Le*(c)	*Mi*	*Pa*	*Qu*	*Rh*
Disturbed	A	43	0	0	1	0	30	1	0	0	0	0	1
Disturbed	A	63	0	0	15	0	97	11	0	0	0	0	0
Undisturbed	A	124	0	0	7	2	151	0	0	0	0	2	6
Undisturbed	A	105	0	2	7	0	117	0	0	0	1	3	6
Disturbed	B	4	0	0	14	0	27	3	0	8	2	0	0
Disturbed	B	5	0	0	4	0	35	0	0	3	0	0	0
Undisturbed	B	91	0	0	4	0	15	2	0	0	0	0	0
Undisturbed	B	57	0	0	5	0	88	5	0	0	1	0	0
Disturbed	C	7	0	0	2	0	3	0	10	0	0	0	0
Disturbed	C	6	0	0	3	0	1	0	180	1	0	0	0
Undisturbed	C	10	0	1	5	0	3	0	0	1	0	0	0
Undisturbed	C	60	1	4	0	0	0	0	10	0	0	0	0
Disturbed	D	69	4	1	1	0	29	0	3	3	0	0	0
Disturbed	D	5	1	0	1	0	47	1	1	5	0	0	0
Undisturbed	D	142	3	6	2	0	6	0	0	0	0	2	0
Undisturbed	D	96	2	7	1	0	2	0	0	0	0	0	0

The key stumbling block is developing a resampling algorithm that preserves in resamples both the non-normality of the data and the fixed nature of the sampling design. Permutation tests, which are exact in some designs [3], are not directly applicable for the example of Table 1 as the null hypothesis (in a test for interaction) does not imply that observations in different treatment groups are exchangeable. Case resampling [2], where we resample rows of the design matrix and response matrix jointly, creates singularities in the design matrix with high probability (each treatment-block combination having a 12% chance of containing no replicates). Residual resampling is a way forward, since it keeps the sampling design fixed in resamples, but it requires identically distributed residuals to be available. What is needed is a general method of calculating residuals for data from any parametric distribution, that will produce approximately independently and identically distributed (iid) residuals under the null hypothesis.

**Fig 1:**
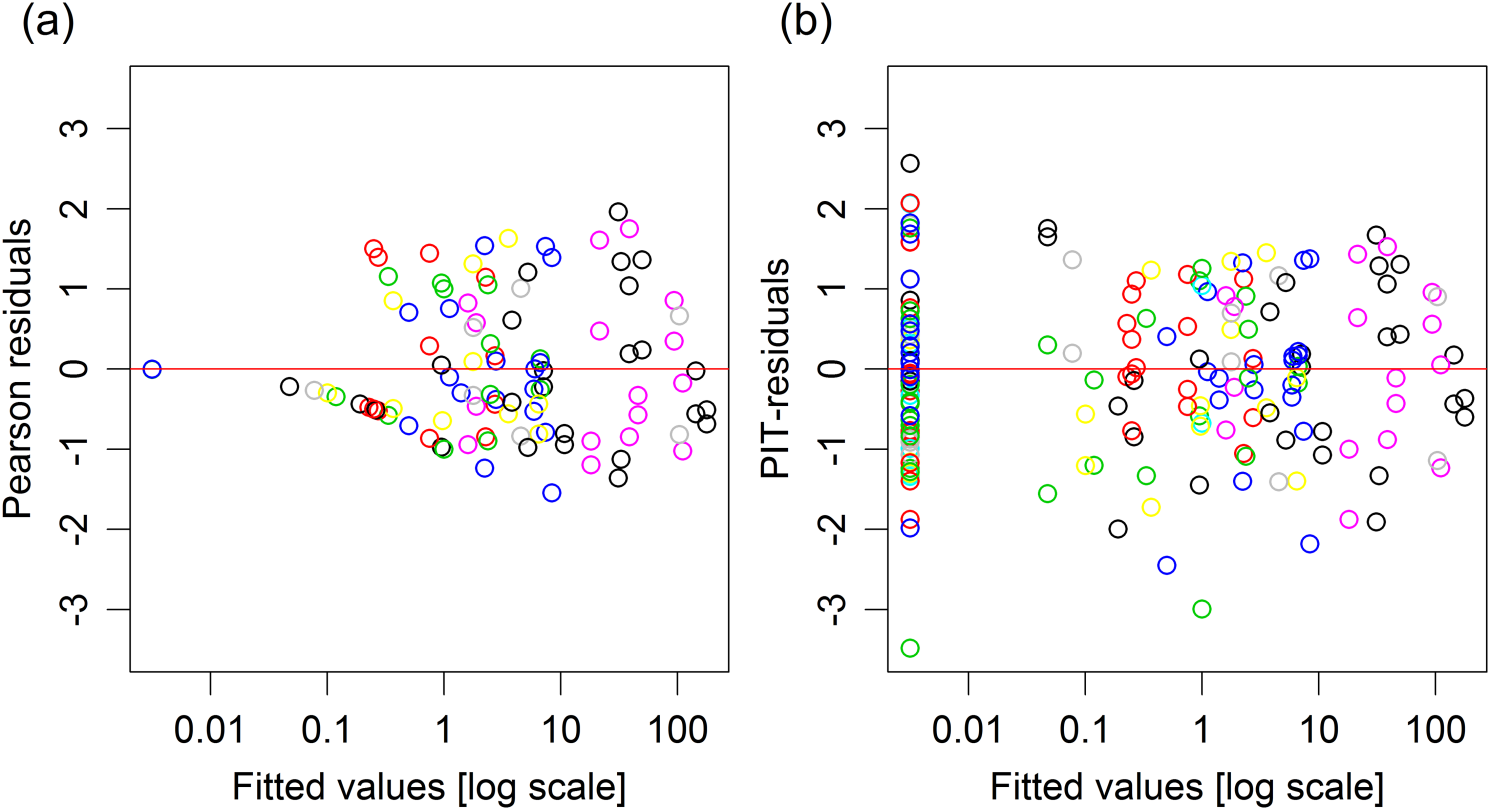
Residual plots for a negative binomial regression model fitted to the copepod data of Table 1, using (a) Pearson residuals; (b) PIT-residuals. Different colours used for different species. Notice that the predominant patterns in (a) are the line of points towards the left (corresponding to zeros) and asymmetry about the horizontal line *y* = 0 (marked in red). These trends, due to the discreteness of the data rather than lack of fit, have been removed in (b) such that the reader can focus on the question of goodness-of-fit.

There is a generally applicable definition of residuals for parametric regression models that can produce approximately iid observations—the probability integral transform (PIT) residual [11]. PIT-residuals have the key property that if the regression model is correct and the true values of parameters are known, they are exactly an iid sample from the standard uniform distribution. In practice, PIT-residuals can only approximately satisfy these properties, because of sampling error estimating parameters, which are usually not known. PIT-residuals, or variants thereof, have hence been proposed primarily for use as diagnostic tools, and referred to by a variety of names, including forecast distribution transformed residuals [11], randomized quantile residuals [12] and universal residuals [13]. A particular part of their appeal is that they have the same distribution (when the model is true) irrespective of the values of model parameters, and irrespective of discreteness in the raw data. Hence, for example, when comparing residual vs fits plots for the data of Table 1, it is difficult to assess model fit using Pearson residuals (Fig 1a), but the model fit appears reasonable upon study of the PIT residuals (Fig 1b).

In this paper we propose the PIT-residual bootstrap, which we refer to for conciseness as the “PIT-trap”. The name is a reference to pit traps, or pitfall traps, a method of sampling invertebrates in ecology that is commonly used to collect data of the form of Table 1. This method can also be understood as a special type of “model-free bootstrap” [14] for discrete, highly multivariate data. The idea behind our method is also very similar to the residual bootstrap proposed by [15], a key distinction being our extension of the method to handle regression models for responses from any distribution, including the discrete case, and our derivation of some key properties of the PIT-trap. One particularly useful property is that standard results from the classical bootstrap apply quite generally to the PIT-trap, including second order accuracy of pivotal test statistics, a benefit not afforded to competing residual resampling approaches [2]. A second useful property, that does apply to any residual resampling approach, is that when applied to multivariate data, the PIT-trap can preserve the correlation in the data without explicitly modelling it via row-resampling. This is the key point of difference as compared to a parametric bootstrap.

First PIT-residuals are reviewed, then the PIT-trap is proposed and some of its key properties discussed, then the approach is applies to the data of Table 1, and simulations are reported which verify some desirable properties of the method.

## PIT-residuals

The key innovation in this paper makes use of probability integral transform (PIT-) residuals, which are reviewed below. PIT-residuals have been used by others to develop related bootstrap algorithms for continuous data [14, 15].

It is well-known that for a univariate, continuous *Y*, which has cumulative distribution function *F*(*y*; ***θ***), U=F(Y;θ)∼U(0,1) where U(0,1) denotes the standard uniform random variable. A multivariate version of this result maps a *p*-variate variable **Y** onto the uniform distribution on the *p*-variate unit cube [16], but in this paper only the univariate version of this result will be used.

This result can be generalized to discrete data as follows [16]:

$$\begin{array}{c} U=Q\;F\left(Y;\theta \right)+\left(1-Q\right)\;F\left(Y^{-};\theta \right)∼U\left(0,1\right) \end{array}$$

where *F*(*y*^−^) denotes the limiting value of *F*(*y*) as *y* is approached from the negative direction, and *Q* is a standard uniform random variable independent of *Y*. The random variable *Q* is introduced to handle the discreteness in the data, by uniformly distributing the probability mass from the point *F*(*y*) across all real values between *F*(*y*) and the previous allowable value of this function *F*(*y*^−^).

The above results can be used to define probability integral transform residuals (PIT-residuals) as follows. Consider a parametric regression model for *Y*, conditional on explanatory variables **x**, which has cumulative distribution function *F*(*y*; ***θ***, **x**). The PIT-residual *u*_*i*_ corresponding to the observation *y*_*i*_ conditional on **x**_*i*_ is defined as follows:

$$\begin{array}{c} u_{i}=\{\begin{array}{cc} F(y_{i};\hat{\theta },x_{i}) & \text{if}\;Y_{i}\;\text{is}\;\text{continuous}\\ q_{i}\;F\left(y_{i};\hat{\theta },x_{i}\right)+\left(1-q_{i}\right)\;F\left(y_{i}^{-};\hat{\theta },x_{i}\right) & \text{if}\;Y_{i}\;\text{is}\;\text{discrete} \end{array} \end{array}$$ (1)

where *q*_*i*_ is an observation from the standard uniform distribution.

While the *u*_*i*_ are typically referred to as residuals [11, 13], they do not behave like residuals in the usual sense—they are centred around a value of 0.5 rather than a value of 0, and are bounded between 0 and 1. To address this the *u*_*i*_ can be mapped onto the standard normal distribution, *z*_*i*_ = Φ^−1^(*u*_*i*_) [12], to improve interpretability (as in Fig 1). Whether or not this is done is irrelevant to the development of the PIT-trap, in the next section.

Note that if $\hat{\theta }\overset{P}{\to }\theta$ then the *u*_*i*_ come from a distribution whose limit is U(0,1), provided that *F*(*y*; ***θ***, **x**_*i*_) has been specified correctly. In this sense, PIT-residuals are pivotal quantities. Further, if the *y*_*i*_ are independent, then the only dependence in the *u*_*i*_ is via $\hat{\theta }$, and this dependence decays to zero as sample size increases. So in large samples the *u*_*i*_ are iid—this is the key property that makes PIT-residuals amenable to bootstrapping.

## The PIT-trap

The fundamental idea of this paper is to use a PIT-residual bootstrap, or PIT-trap, as a basis for inference. The idea of a residual resampling technique that makes use of the probability integral transform has been proposed previously in the context of survival analysis [15, 17]. [15] considered Cox proportional hazard models for univariate data, and later generalized his algorithm to multivariate survival analysis [17] by making use of the marginal cumulative distribution and row resampling. We do the same in the below, but apply the technique more generally beyond proportional hazard models, and study some of its theoretical properties. As [17] note, the idea of making inferences based on models for the marginal distribution, but which are robust to correlation in responses, is very much in the spirit of generalized estimating equations [1] methodology, previously adapted to problems such as the analysis of Table 1 [10].

Another related idea is the “model-free” approach [14, 18] to fitting and prediction, based on analysis of residuals and searching for transformations to “iid-ness” that imply particular models for the observed data. [14] proposed a “model-free bootstrap”, based on resampling transformed quantities that play the role of PIT-residuals, for continuously distributed data. This is referred to as model-free on the grounds that it is motivated via transformation of the response to iid-ness rather than from fitting a given model to data. Thus PIT-residual resampling, as proposed below, could be understood as a type of model-free bootstrap, where the PIT-residuals are considered as the data transformation to “iid-ness”. While the model-free bootstrap of [14] required the response to be continuous, such that there was a one-to-one transformation between response and residuals, in the discrete data case [14] advocated a “limit model-free bootstrap”, related to the parametric bootstrap, to be discussed later. Below we propose an alternative but related approach appropriate for discrete and highly multivariate data. Specifically, discreteness will be handled by using PIT-residuals defined with “jittering” via *Q*, such that algorithms along the lines of [15] and [14] can be extended directly to discrete data. Further, multivariate data will be handled using block resampling so that we are not required to model the correlation structure in the data—a useful property when analysing data such as in Table 1, where data are not very informative about the correlation structure, especially given the large number of response relative to the number of observations.

The PIT-residual bootstrap, or PIT-trap, can be applied whenever we observe a *n* × *p* matrix of responses $y={\left(y_{1}^{T},\ldots ,y_{n}^{T}\right)}^{T}$ for which marginal distribution functions are available for each variable in **y**_*i*_ = (*y*_*i*1_, …, *y*_*ip*_). Denote as *F*(*y*; ***θ***_*j*_, **x**_*i*_) the distribution of *y*_*ij*_ that is marginal with respect to *y*_*ik*_, *k* ≠ *j*, but conditional on covariates **x**_*i*_. The focus in this paper will be on the case where the *p*-variate observations **y**_*i*_ are independent of each other, but dependence can also be handled if the *F*(**y**; ***θ***_*j*_, **x**_*i*_) specify the *conditional* distribution of **y**_*i*_ given previous responses **y**_1_, …, **y**_*i*−1_ as in [13]. Each response observation *y*_*ij*_ in the cluster **y**_*i*_ has been assumed in our notation to be related to the same set of covariates **x**_*i*_, as that is most relevant to our situation, but if this assumption were relaxed the below results would still apply.

The PIT-trap is simply a bootstrap method that resamples PIT-residuals {**u**_1_, …,**u**_*n*_}, then uses these to construct resampled values $\left\{y_{1}^{*},\ldots ,y_{n}^{*}\right\}$ by inverting the cumulative distribution function. The PIT-trap algorithm computes resamples T* to approximate the sampling distribution of some statistic *T* = *g*(**y**) as follows:

1. Estimate $\hat{\theta }$ by fitting the regression model to the observed **y**.
2. Generate an *n* × *p* matrix, **q**, of independent random values from the standard uniform distribution.
3. Calculate an *n* × *p* matrix of PIT residuals $u={\left(u_{1}^{T},\ldots ,u_{n}^{T}\right)}^{T}$ by applying Eq (1) element-wise to {**y**, **q**}. Optionally, each column can be centered and rescaled (see below).
4. For *b* = 1 … *B*:
  - Resample with replacement the *n* vectors {**u**_1_, …, **u**_*n*_}, to obtain $\left\{u_{1}^{*},\ldots ,u_{n}^{*}\right\}$.
  - Calculate resampled *p*-variate observations $\left\{y_{1}^{*},\ldots ,y_{n}^{*}\right\}$ by solving for each $y_{ij}^{*}$ as a function of $u_{ij}^{*}$ (the *j*th elements of $y_{i}^{*}$ and $u_{i}^{*}$ respectively):
    $$\begin{array}{c} \{\begin{array}{ll} u_{ij}^{*}=F\left(y_{ij}^{*};{\hat{\theta }}_{j},x_{i}\right) & \text{if }Y_{ij}\text{ is continuous}\\ F\left({\left(y_{ij}^{*}\right)}^{-};{\hat{\theta }}_{j},x_{i}\right)<u_{ij}^{*}<F\left(y_{ij}^{*};{\hat{\theta }}_{j},x_{i}\right) & \text{if }Y_{ij}\text{ is discrete} \end{array} \end{array}$$
  - Compute the required statistic, T* = *g*(y*).

If data are discrete, then some randomness has been introduced into residual calculation by **q**. This can be accounted for in the PIT-trap algorithm by recalculating **q** and **u** for each resample, i.e. moving steps 2-3 inside the loop at step 4 rather than leaving them outside, at some (usually small) computational cost.

This algorithm is especially suited to situations where we have a reasonable idea of the nature of the marginal distribution of the *y*_*ij*_, but relatively little knowledge of the correlation within clusters. If the marginal distribution $F(y_{ij};{\hat{\theta }}_{j},x_{i})$ is correct then PIT-residuals may be correlated across response variables but they will (asymptotically) be marginally standard uniform. By resampling rows (clusters) of data $\left\{u_{1}^{*},\ldots ,u_{n}^{*}\right\}$ as proposed, the correlation structure in the *p*-variate observations is preserved in resamples and subsequently accounted for in inferences.

In small samples, we have found improved performance when centering and rescaling residuals, as in [19] and [20]. The method used was to map them onto the standard normal distribution (Φ^−1^(*u*_*ij*_)) and divide by their sample standard deviation, denoted as *s*_Φ^−1^(*u*_*j*_)_. That is, we calculated rescaled residuals ${\tilde{u}}_{ij}$ to satisfy

$$\begin{array}{c} \Phi^{-1}\left({\tilde{u}}_{ij}\right)=\Phi^{-1}\left(u_{ij}\right)/s_{\Phi^{-1}\left(u_{j}\right)} \end{array}$$

This rescaling can be understood as an empirical correction for the fact that the model typically overfits the data, thus underestimates the magnitude of residuals. This is especially the case in small samples and the effect tends to vanish as sample size increases.

### Properties of the PIT-trap

In the below we will derive some attractive properties of the PIT-trap algorithm.

**Theorem 1** *Consider a PIT-trap sample of* **Y**, *where the* (*i*, *j*)*th PIT-trap value* $Y_{ij}^{*}$ *is computed using a plug-in estimate of the marginal distribution of* *Y*_*ij*_, $F(y;{\hat{\theta }}_{j},x_{i})$, *and the true marginal distribution of* *Y*_*ij*_ is *F*(*y*; ***θ***_*j*_, **x**_*i*_). *Assume* *F*(*y*; ***θ***_*j*_, **x**_*i*_) *is twice differentiable with respect to* ***θ***_*j*_.

*For each j*, *if* ${\hat{\theta }}_{j}$ *is a* $\sqrt{n}$-*consistent estimator of* ***θ***_*j*_, *then*:

$$\begin{array}{c} P_{*}\left(Y_{ij}^{*}\leq y\right)=F\left(y;\theta_{j},x_{i}\right)+O_{p}\left(n^{-1/2}\right) \end{array}$$

*where* $P_{*}\left(A\right)$ *denotes the probability of* A *from repeated PIT-trapping*.

The proof, and indeed all proofs, can be found in on-line supplementary material (S1 File).

Theorem 1 shows that when applied to a large data set, the distribution of a PIT-trapped observation $y_{ij}^{*}$ approximates the true marginal distribution of the corresponding original observation *y*_*ij*_, converging to the target marginal distribution asymptotically. The conditions under which this result was derived—a known parametric form for the marginal distribution *F*(*y*; ***θ***_*j*_, **x**_*i*_), and a $\sqrt{n}$-consistent estimate of the parameter $\hat{\theta }$—considerably relaxes the conditions previously required for residual resampling to be applicable, to the point where the method can readily be applied to most common parametric regression models, including the important case of generalized linear models for discrete data.

Theorem 1 can be generalized to handle misspecification of the marginal distribution, if the “working” marginal distribution leads to pivotal PIT-residuals. This follows since the proof to Theorem 1 does not use the fact that the PIT-residuals *u*_*ij*_ come from a standard uniform distribution, it only requires them to be iid.

The PIT-trap, when applied to clusters of correlated data, will preserve the correlation in the data, as in the following theorem.

**Theorem 2** *Consider p-variate PIT-trap residuals* $U_{i}^{*}$, *i* = 1, …, *n*, *obtained by resampling PIT-residuals with replacement. Let* $\hat{\Sigma }$ *be the sample variance-covariance matrix of PIT-residuals*, $\hat{\Sigma }=\frac{1}{n}\sum_{i=1}^{n}{\left(u_{i}-{\bar{u}}_{i}\right)}^{T}\left(u_{i}-{\bar{u}}_{i}\right)$. *Then*:

$$\begin{array}{c} var_{*}\left(U_{i}^{*}\right)=\hat{\Sigma } \end{array}$$

where var_*_(⋅) *denotes the variance-covariance matrix under repeated PIT-trapping*.

The proof is straightforward and is omitted.

Note that Theorem 2 does not necessarily imply that $var\left(U_{i}^{*}\right)\to var\left(U_{i}\right)$ because it may not be the case that the **U**_*i*_ share the same variance-covariance matrix **Σ**. If they do not, then the **U**_*i*_ are not identically distributed and the PIT-trap might not have desirable properties for multivariate inference. On the other hand, when the **U**_*i*_ are identically distributed, we can show the following result.

**Theorem 3** *Let T* = *g*(**Y**) *be an asymptotically standard normal statistic calculated from some multivariate sample* **Y** *characterized by its marginal distributions F*(*y*; ***θ***_*j*_, **x**_*i*_) *and the variance-covariance matrix of PIT-residuals*, *var*(**U**_*i*_) = **Σ**. Let T* = *g*(Y*) *be the same statistic calculated from the PIT-trap sample* Y* *using* $F(y;{\hat{\theta }}_{j},x_{i})$. *Assume* **Y** *and g*(⋅) *are such that T admits an Edgeworth expansion. If* ${\hat{\theta }}_{j}$ *is* $\sqrt{n}$-*consistent for each* *j* *then*

$$\begin{array}{c} P_{*}\left(T^{*}\leq t\right)=P\left(T\leq t\right)+O_{p}\left(n^{-1}\right) \end{array}$$

$$\begin{array}{c} P_{*}\left(-t\leq T^{*}\leq t\right)=P\left(-t\leq T\leq t\right)+O_{p}\left(n^{-3/2}\right) \end{array}$$

This theorem parallels standard bootstrap results [21] and can be proved in a similar way, using Edgeworth expansions (with some modification to handle the discrete case).

The conditions under which *T* admits an Edgeworth expansion, required for Theorem 3, can be found in [21], but briefly, **Y** must satisfy Cramér’s condition, the first four moments of **Y** must be finite, and *g* must be able to be expressed as a smooth function of means of independent random variables (in the sense that it is four times differentiable in the neighbourhood of the mean).

We use simulation later to consider the question of how robust the performance of the PIT-trap is to settings where the conditions of Theorem 3 are not satisfied—specifically, we are concerned about situations where the distributional assumptions are mildly misspecified.

The requirement that the joint distribution of **Y** be characterized by its marginal distributions and the variance-covariance matrix of PIT-residuals **Σ** is not unlike working assumptions often made in the generalized estimating equations literature [1], but it also has an interesting connection to the literature on copula models [22]. Copula models are specified via a similar structure to the PIT-trap, and one way to view the PIT-trap is as a bootstrap method for a type of copula model. In the simulation section we use a copula model to generate correlated data in order to assess the performance of the PIT-trap.

### Relation to other bootstrap methods

Beyond [14, 15, 17], the PIT-trap has relationships to other bootstrap methods, most closely, the parametric bootstrap [2]. Roughly speaking, the PIT-trap can be understood as a compromise between the parametric bootstrap and residual resampling, inheriting the attractive features of each method—the ability to generate data (approximately) from *F*(*y*; ***θ***_*j*_, **x**_*i*_) is inherited from the former, and the ability to preserve correlation in clustered data is inherited from the latter.

The parametric bootstrap follows a very similar algorithm to the PIT-trap, the key difference being that it obtains PIT-residuals ($u_{ij}^{*}$) by simulating them instead of resampling them. That is, the parametric bootstrap can be understood as replacing step 4a of the PIT-trap algorithm as follows:

(a) Generate $u_{ij}^{*}$ from U(0,1).

Hence the inverse transformation of step 4b generates samples directly from $F(y;{\hat{\theta }}_{j},x_{i})$. (Step 3 is redundant for the parametric bootstrap and could be removed.)

[14] proposed the limit model-free bootstrap, and advocated the approach for bootstrapping discrete data. The procedure requires as a starting point a transformation *G*(**y**_*i*_) of the data to “iid-ness”. Then one simulates standard uniform values $u_{ij}^{*}$ and obtains bootstrapped values as $y_{i}^{*}=G^{-1}\left(u_{i}^{*}\right)$. Note that, by the probability integral transform, this generates independent data with distribution function *G*(**y**_*i*_), hence the limit model-free bootstrap can be understood as a parametric bootstrap assuming *G*(**y**_*i*_).

In the case of multivariate or clustered data, step 4a of a parametric bootstrap involves simulating vectors of $u_{i}^{*}$ with covariance matrix $\hat{\Sigma }$, but where each element is marginally U(0,1). $\hat{\Sigma }$ should be a consistent estimate of Σ.

A key point of difference between the parametric bootstrap and the PIT-trap is in their assumptions. The PIT-trap resamples *u*_*ij*_ under the assumption that the PIT-residuals *u*_*ij*_ are iid for *i* = 1, …, *n* in the limit as *n* → ∞. An important situation where this assumption can be satisfied is when the marginal distribution of the *y*_*ij*_ has been correctly specified and PIT-residuals share a common correlation structure. The parametric bootstrap, in contrast, requires the full joint distribution of data to be specified correctly—i.e. the correlation structure must be known in order to simulate $u_{ij}^{*}$ with the required correlation structure. Hence the PIT-trap is applicable in some settings where the parametric bootstrap is not—in particular, generalized estimating equations, for which a joint distribution is not specified, and situations where data are clustered but the precise form of within-cluster correlation is not well understood. An important example of where the within-cluster correlation is not well understood is when **y** is high-dimensional [10], or more generally, when *p* is not small compared to *n*.

A different way to view the PIT-trap is as a special type of residual resampling, indeed it inherits some advantages from residual resampling—in particular, the ability to resample clustered data in such a way that any within-cluster correlation between residuals can be preserved. Also, residual resampling methods are often advocated in the regression context [2, 21] because they preserve the conditioning on **x**_*i*_. The key issue when implementing residual resampling for non-Gaussian regression models, most commonly using Pearson and deviance residuals [2, 23], has been that they do not result in pivotal quantities in general. Subsequent residual-resampled data Y* does not adequately approximate the sampling distribution of **Y** in general. For discrete and sparse data, as in Table 1, subsequent bootstrap samples would deviate conspicuously from the desired distribution, some values being non-integer, negative or even undefined. Correcting for this issue could introduce considerable bias. In contrast, the PIT-trap circumvents these difficulties, provided that the parametric form of the marginal distribution of data is known.

A final set of relations of interest link the PIT-trap to classical resampling methods for models with iid errors. Consider first the model *y*_*ij*_ = *μ*_*ij*_ + *ϵ*_*ij*_, where the *μ*_*ij*_ are fixed and the random errors *ϵ*_*ij*_ are parameterized by their standard deviation *σ* only, linear regression being an important special case. For such models, raw residuals $r_{ij}=y_{ij}-{\hat{\mu }}_{ij}$ are monotonically related to PIT-residuals:

$$\begin{array}{c} u_{ij}=F\left(y_{ij};{\hat{\mu }}_{ij},\hat{\sigma }\right)=F\left(r_{ij};0,\hat{\sigma }\right) \end{array}$$

This monotonicity implies that bootstrapping PIT-residuals is equivalent to the standard residual resampling approach where raw residuals are bootstrapped [2], if one assumes errors are iid. Now consider the situation where we wish to test the null hypothesis that all observations are iid. In this case, and by a similar argument, the PIT-trap reduces to resampling the **y**_*i*_ with replacement. Further, if resampling PIT-residuals without replacement, this would reduce to the usual permutation test [3]. Hence many classical resampling methods can be understood as special cases of the PIT-trap, the key innovation of the PIT-trap being its ability to extend these well-known resampling methods to parametric modeling where errors are no longer iid.

## Practical application

In this section the PIT-trap will be applied to the data of Table 1 [8]. This data set consists of counts of the abundance of twelve species of copepod (small crustaceans) in 16 sites. The study was conducted to explore the effect of crab disturbance on copepod communities on intertidal sandflats on the Tasman Peninsular, Australia. A randomized blocks design was used where two disturbed and two undisturbed sites were sampled at each of four sites (“blocks”).

The purpose of analysis was to test for evidence of an effect of disturbance (treatment effect), and whether the effect was different at different sites (block×treatment interaction). When testing for interaction, difficulties arise for resampling-based hypothesis testing because observations are not exchangeable under the null hypothesis, and instead residual resampling has been proposed [3].

Three important properties of the data are evident in Table 1. Firstly, there are many zeros in the data, because not every species is observed in every site. Secondly, abundance appears to be strongly right-skewed. Each of these features is problematic for most types of residuals that have previously been defined [2, 23]. Thirdly, the number of variables (*p* = 12) is not small compared to the number of observations (*n* = 16), which motivates the use of resampling for inference as in [9, 10].

Negative binomial regression models [24] were fitted to the data, to account for overdispersion in the counts. Let *Y*_*ijkl*_ be the abundance of the *l*th replicate (*l* ∈ {1, 2}) at site *k* ∈ {1, 2, 3, 4} of species *j* ∈ {1, …, 12} in treatment *i* ∈ {1, 2}. We assumed *Y*_*ijkl*_ have a negative binomial marginal distribution with mean *μ*_*ijk*_ satisfying:

$$\begin{array}{c} \log\left(\mu_{ijk}\right)=\alpha_{0j}+\alpha_{ij}+\beta_{kj}+\gamma_{ijk} \end{array}$$ (2)

where the *α*_*ij*_, *β*_*kj*_ and *γ*_*ijk*_ respectively represent treatment, block and treatment×block effects for the *j*th species, and for identifiability, each of these terms is zero whenever it is indexed by an *i* or *k* which equals one.

The variance was assumed to be a quadratic function of the mean:

$$\begin{array}{c} Var\left(Y_{ijkl}\right)=\mu_{ijk}+\psi_{j}\mu_{ijk}^{2}. \end{array}$$ (3)

and a residual plot (Fig 1b) suggested this model accounted for overdispersion in the data reasonably well.

Model parameters were estimated separately for each species via maximum likelihood. This corresponds to a working assumption of independence across species, but we expect that there is in fact correlation across species, despite it not being explicitly modelled in the above.

We tested the hypothesis of no interaction:

$$\begin{array}{c} H_{0}:\gamma_{ijk}=0\;\forall i,k\;H_{a}:\text{otherwise} \end{array}$$

using a score statistic based on a ridge-regularized estimate of the correlation matrix of residuals [10]. This approach incorporates correlation between species into the test statistic, using linear shrinkage towards an identity matrix in order to obtain a more numerically stable statistic which has better properties [9, 10]. We assessed the significance of this test statistic using 1000 resamples from each of a residual bootstrap using Pearson residuals, the PIT-trap with negative binomial marginals, and a parametric bootstrap which simulated from a copula model with an unstructured correlation matrix, and again assumed negative binomial marginals. The copula was fitted in a two-step approach, using negative binomial regression again to estimate parameters in the marginal distribution, and a ridge-regularised estimate of the correlation matrix [10] of PIT-residuals mapped onto the standard normal, Φ^−1^(*u*_*ij*_)

The observed test statistic for a test for no interaction was 40.99, and the bootstrap estimates of the *P*-value for the PIT-trap, parametric bootstrap and Pearson residual bootstrap were (to three decimal places) 0.039, 0.046, and 0.014, respectively. The difference between the Pearson result and the other two is larger than would be expected by Monte Carlo error, and while all suggest some evidence of an interaction effect, only marginally so for the PIT-trap and parametric bootstrap.

We have developed freely available software in the R package mvabund [25], indeed our PIT-trap and Pearson residual bootstrap results can be easily replicated using the following code:

data(Tasmania)

attach(Tasmania)

abund = mvabund(copepods)

ftMain = manyglm(abund~block+treatment, family = “negative.binomial”)

plot(ftMain)

ftInter = manyglm(abund~block*treatment, family = “negative.binomial”)

anova(ftMain, ftInter, cor.type = “shrink”, test = “score”, p.uni = “adjust”, resamp=***)

where *** is chosen to be “pit.trap” or “residual” (Pearson residuals).

## Simulations

We conducted two simulation studies to investigate the small sample properties of the PIT-trap, in comparison to its immediate competitors.

### Logistic regression

First we studied logistic regression in small to moderate samples. We compared the properties of likelihood ratio tests where the null distribution was estimated using the PIT-trap, the Pearson residual bootstrap, the parametric bootstrap, case resampling, and the usual chi-squared approximation from classical statistics.

The sampling design, inspired by Table 1, was a randomized blocks design with four blocks, two treatments, and balanced sampling. We generated Bernoulli random variables with the mean in the *i*th treatment and the *k*th block given by:

$$\begin{array}{c} \log(\frac{\mu_{ik}}{1-\mu_{ik}})=\alpha_{0}+\alpha_{i}+\beta_{k}+\gamma_{ik} \end{array}$$ (4)

where as previously the *α*_*i*_, *β*_*k*_ and *γ*_*ik*_ respectively represent treatment, block and treatment×block effects, and for identifiability, each of these terms is zero whenever it is indexed by an *i* or *k* which equals one. We tested for no interaction:

$$\begin{array}{c} H_{0}:\gamma_{ik}=0\;\forall i,k\;H_{a}:\text{otherwise} \end{array}$$

This hypothesis is of biological interest, while also being difficult to resample data from, since response observations *Y*_*i*_ are not exchangeable under *H*_0_.

We simulated 1000 data sets such that (*α*_0_, *α*_2_, *β*_2_, *β*_3_, *β*_4_, *γ*_22_, *γ*_23_, *γ*_24_) = (−1, 1, 0, −1, 1, 0, 0, 0) and varied the number of replicates such that total sample size ranged from 16 to 128. Each bootstrap test was conducted using 1000 bootstrap samples, and we compared Type I error at the 0.05 significance level.

**Fig 2:**
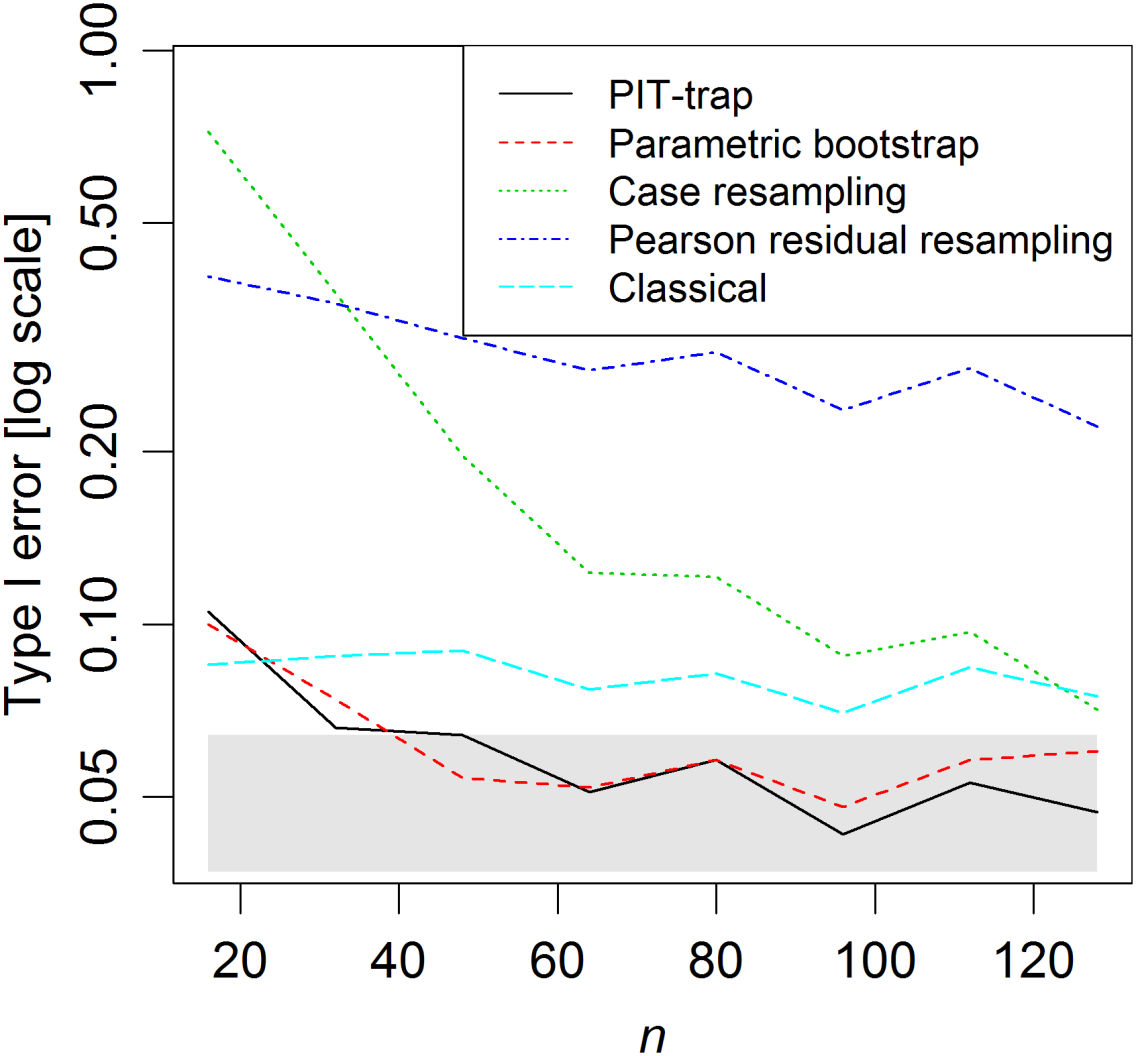
Type I error of different resampling methods in logistic regression simulations varying *n*. The shaded grey region represents a 95% confidence band around the nominal Type I error rate of 0.05. Note that both the PIT-trap and parametric regression performed well for *n* ≥ 32, the Pearson residual bootstrap was very poor for all *n*, and (for small-moderate *n*) classical tests via the *χ*^2^ distribution had inflated Type I error, motivating the use of resampling.

The PIT-trap and parametric bootstrap converged rapidly to nominal Type I error levels (Fig 2), as expected from Theorem 3, with close to nominal levels when there were as few as four replicates per treatment group. The classical test for interaction, comparing the likelihood ratio statistic to a $\chi_{3}^{2}$ null distribution, was too liberal for small-moderate sample sizes, and was relatively slow to converge to nominal levels. Hence these results provide some motivation for using the PIT-trap or parametric bootstrap to test hypotheses concerning logistic regression parameters using small-moderate samples.

A striking pattern in results (Fig 2) was that the Pearson residual bootstrap was grossly inadequate, with Type I error always in excess of four times the nominal level, and not converging to the nominal level as *n* increased. As explained previously, Pearson residual resamples (Y*) do not have the correct marginal distribution—resampled values typically are not integers and are not always between 0 and 1. The latter we believe is the greater problem, because it is necessary in test statistic construction to truncate data to the unit interval [0, 1], which introduces bias and changes the mean-variance relationship of the bootstrap samples.

Case resampling also had very highly inflated Type I error at small sample sizes, but converged towards correct values as *n* increased. Case resampling treats the number of replicates per treatment-block combination as random, which leads to unbalanced designs in resamples, with some treatment-block combinations often empty. This is the likely reason for the poor small-sample performance, with randomness in the design introducing additional uncertainty, considerably so for small *n*.

### Multivariate counts

A second simulation generated multivariate count data to mimic the properties of the data of Table 1, looking at the effect of increasing dimension of the data as well as increasing sample size.

Correlated, overdispersed count data were generated via two methods—using a copula model, and a Poisson lognormal model. In the copula approach, we generated **z**_*i*_ ∼ *MVN*(**0**, **R**) for some correlation matrix **R**, then *u*_*ij*_ = Φ(*z*_*ij*_) and *Y*_*ijkl*_ = *F*^−1^(*u*_*ij*_; *μ*_*ijk*_, *ψ*_*j*_) where Φ(*x*) and *F*(*x*; ⋅, ⋅) are the cumulative distribution functions of the standard normal and negative binomial distribution, respectively, and *μ*_*ijk*_ is defined in Eq 2. Hence copula data had constant *cov*(**U**_*i*_), as was required for Theorem 3. The Poisson lognormal model simulated counts as *Y*_*ijkl*_ ∼ *Poisson*(*m*_*ijk*_) and

$$\begin{array}{c} \log\left(m_{ijk}\right)=\alpha_{0j}-0.5\sigma_{j}^{2}+\alpha_{ij}+\beta_{kj}+\gamma_{ijk}+z_{ij} \end{array}$$

where *z*_*ij*_ was the *j*th element of **z**_*i*_ ∼ *MVN*(**0**, Σ_*d*_**R**Σ_*d*_) and *σ*_*j*_ played the role of *ψ*_*j*_ in controlling the extent of overdispersion. The values of *σ*_*j*_ for the *p* response variables were stored in the diagonal matrix Σ_*d*_. This model maintained the same mean model and mean-variance relationship as for the negative binomial copula model, but data were no longer marginally negative binomial and no longer had constant *cov*(**U**_*i*_). Hence this second simulation gives some insight into the robustness of the PIT-trap to (modest) violations of assumptions.

The values for slope parameters to be used in simulations were taken from the fit of the null (main effects) model to the sample data of Table 1. To look at the effect of average species abundance on performance, we multiplied the matrix of the means ***μ*** by a factor *δ* ∈ {1, 2.5, 5}. The correlation matrix was set using an AR(1) structure with the autocorrelation parameter *ρ* ∈ {0.5, 0.6, 0.7, 0.8, 0.9} to look at the effect of strength of correlation structure on performance. Results were similar across different values of *ρ* so we only report *ρ* = 0.7 here. We varied the sample size *n* ∈ {16, 32, 64, 96, 128, 160} and number of variables *p* ∈ {12, 24, 36, 48, 60} by replicating the design matrix and the matrix of means ***μ*** as required.

We used the same testing procedure here as in the practical application described previously: fitting negative binomial distributions to each species, then constructing a score statistic which estimates correlation between variables using a ridge-regularized correlation matrix [10]. We compared results when significance of this statistic was assessed using Pearson residual resampling, the PIT-trap, and the parametric bootstrap assuming either an unstructured correlation matrix or incorrectly assuming an exchangeable correlation structure. The latter choice looks at the question of robustness of the parametric bootstrap to misspecification of the correlation structure.

As before, for each of 1000 sample data sets, 1000 resamples were used to estimate the *P*-value of a test for interaction, and the Type I error rate at the 0.05 level was recorded. This was very computationally intensive, taking a total of over a year of computation time on 2.8 GHz processors.

**Fig 3:**
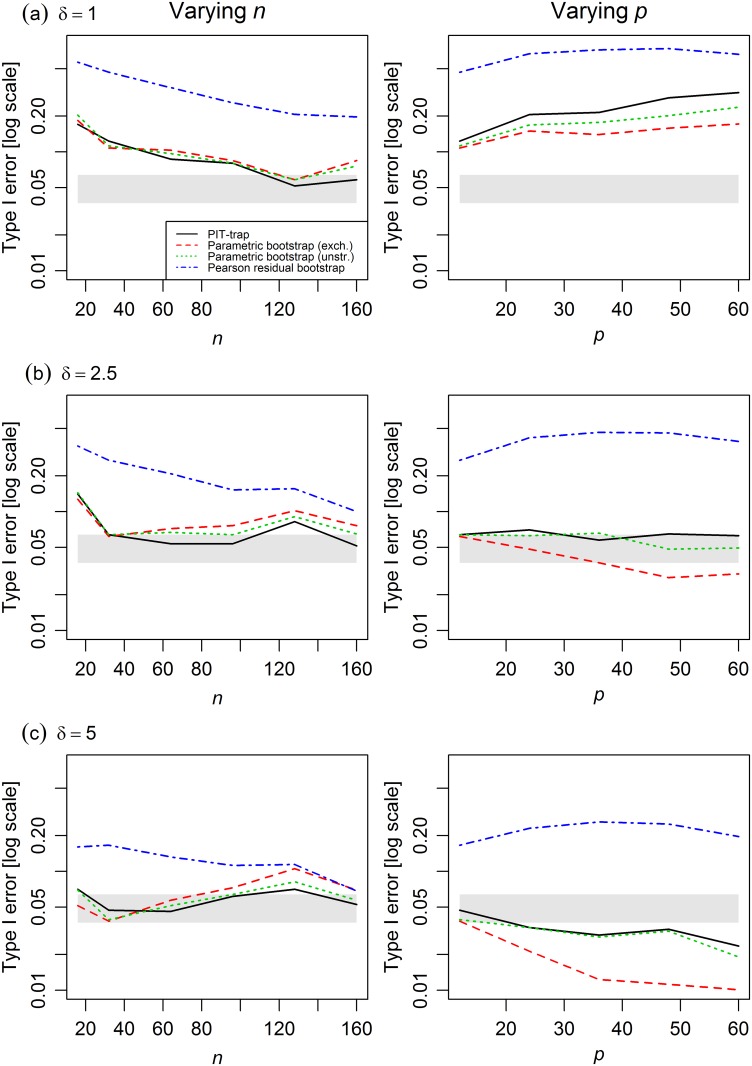
Type I error of different resampling methods in multivariate count simulations from a copula model varying *n* (left) and *p* (right). Mean abundances were manipulated by multiplying the matrix of the means ***μ*** by a factor (a) *δ* = 1, (b) *δ* = 2.5, (c) *δ* = 5. The shaded grey region represents a 95% confidence band around the nominal Type I error rate of 0.05. The PIT-trap performed reasonably well in all contexts, although for low abundances it deviated from nominal levels for small *n* and large *p*. The parametric bootstrap also performed well, provided that the correlation structure was taken to be unstructured, it was less reliable if incorrectly assuming an exchangeable correlation structure.

Type I error rates of the PIT-trap approached nominal levels as *n* increased (Fig 3), converged faster for larger mean abundances (Fig 3b and 3c), and remained close to nominal levels as *n* increased. Problems arose in simulations with low abundances (Fig 3a). When abundance was low and sample size small, all tests had inflated Type I error, although this settled down at larger sample sizes. Further, as *p* increased, all tests had inflated Type I error increasing with *p*, if abundance was low. For medium or high abundance this problem did not seem to arise for the PIT-trap.

The PIT-trap seemed to perform better than alternatives. The Pearson residual bootstrap often had highly inflated Type I error, emphasising the costs of bootstrapping quantities that are not pivotal. The parametric bootstrap became problematic if the correlation structure was not correctly specified. When incorrectly assuming an exchangeable correlation structure, it became too liberal as *n* increased (Fig 3, left), and was highly conservative when *p* was not small for large mean abundances (Fig 3c, right). Using an unstructured correlation matrix in combination with the parametric bootstrap performed about as well as the PIT-trap in most cases.

**Fig 4:**
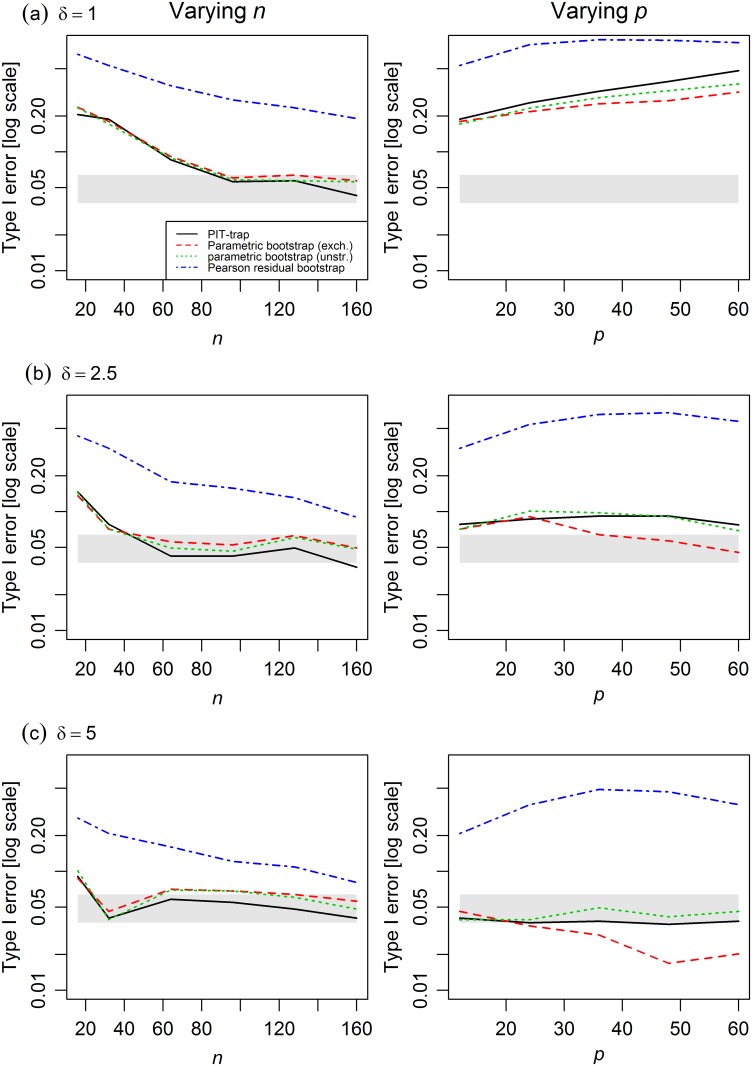
Type I error of different resampling methods in multivariate count simulations from a lognormal-Poisson model, varying *n* (left) and *p* (right). Mean abundances were manipulated by multiplying the matrix of the means ***μ*** by a factor (a) *δ* = 1, (b) *δ* = 2.5, (c) *δ* = 5. The shaded grey region represents a 95% confidence band around the nominal Type I error rate of 0.05. The PIT-trap performed reasonably well in simulations varying *n*, but became inflated at large *p* because of failure of model assumptions, for low-medium abundances (a-b). The parametric bootstrap, assuming an unstructured correlation matrix, performed similarly.

Poisson-lognormal simulations suggested that under violations of the underlying data model, the PIT-trap maintained close to nominal Type I error rates as *n* increased, but Type I error became noticeably inflated at large *p* when mean abundances were low (Fig 4). There seemed to be little difference between the PIT-trap and parametric bootstrap in robustness to violation of underlying model assumptions.

## Discussion

In this paper we have described a very general residual resampling approach, based on probability integral transform, which we refer to as the PIT-trap. This can be understood as a special case of the model-free bootstrap [14], adapted to the problem of discrete, highly multivariate data. The method was demonstrated by theory and simulation to perform well even in a very challenging situation arising in ecology, where data were sparse, overdispersed, and high dimensional. Simulations suggest the method can perform reasonably well when *p* > *n*, and under mild forms of model misspecification, although problems can arise when both of these elements may be present.

The PIT-trap is most closely related to the parametric bootstrap, and in simulations, these two methods behaved similarly (Figs 2–4). A key distinction however is that the PIT-trap only requires knowledge of the marginal distribution of data. In contrast, the parametric bootstrap requires knowledge of the joint distribution, and can perform poorly when assumptions of correlation structure are incorrect. Further, one might expect the PIT-trap to have greater robustness to failure of assumptions in the marginal model (see on-line Appendix, Lemma 2), although this was not borne out in simulations (Fig 4).

Because it is a residual resampling method, the PIT-trap can be used to model clustered observations without explicitly specifying a model for how they are correlated. This is somewhat analogous to the setup under which the generalized estimating equations method was derived [1], and indeed the PIT-trap is readily applicable to problems where generalized estimating equations are used. Another important application, and the one which inspired this work, is the analysis of discrete multivariate data in ecology, when *p* may not be sufficiently small compared to *n* to adequately estimate the correlation structure (Table 1). However simulations suggest that when *p* is too large compared to *n* the PIT-trap might not reliably maintain nominal levels. This is likely to arise in part because the PIT-trap will become increasingly sensitive to assumption violations in the marginal model as *p* increases (Fig 4). But it is also likely to arise in part because errors in the PIT-trap distribution accumulate across response variables, so for example the asymptotic one-tailed approximation of Theorem 3 can be written as a function of *p* as well as *n* as *O*_*p*_(*pn*^−1^). This quantity is not negligible when *p* is large.

PIT-residuals are pivotal measures of the agreement between observed and fitted values for any parametric regression model, and their pivotal nature is a particularly useful property. It is this property that makes them so useful in diagnostic tools [12, Fig 1b], and in this paper, this property has been exploited to develop a very general residual resampling scheme with desirable properties. We speculate that there may be other opportunities to improve methodology for parametric regression modelling via PIT-residuals.

## Supporting information

S1 File Proofs of theorems. (PDF)
