# Supplementary material for: The PIT-trap—A “model-free” bootstrap procedure for inference about regression models with discrete, multivariate responses
Source: PLoS One. 2017 Jul 24;12(7):e0181790. doi: 10.1371/journal.pone.0181790 (PMC5524334; doi:10.1371/journal.pone.0181790)
Supplement: S1 File — (PDF) [file pone.0181790.s001.pdf]

The PIT-trap – a general bootstrap procedure for inference about regression models with non-normal response

David I. Warton, Loic Thibaut and Yi A. Wang

S1 FILE. PROOFS OF THEOREMS

We will first prove two lemmas relating to the situation where the cumulative distribution used for the PIT-trap has been misspecified, but in such a way that PIT-residuals remain identically distributed.

Lemma 1. *Let* $U = F(Y)Q + F(Y^-)(1-Q)$ *be the probability integral transform but where the cumulative distribution* $F(y)$ *may have been misspecified, and the true distribution function is* $G(y) = h\{F(y)\}$ *for some function* $h(\cdot)$.

Then

$$P(U \leq u) = h(u)$$

Proof. For simplicity we will consider the continuous case only, the proof follows via a similar method in the discrete case. Let $u = F(y)$ be the observed value of the probability integral transform residual. Then:

$$P(U \leq u) = P\{F(Y) \leq F(y)\} = P(Y \leq y) = G(y) = h\{F(y)\} = h(u)$$

□

Lemma 1 is used directly in the proof of Lemma 2 below.

Lemma 2. *Consider a set of* n *random variables* $Y_1, \ldots, Y_n$ *with distribution function* $G_i(y)$ *for* Y_i. *A PIT-trap sample* $Y_1^*, \ldots, Y_n^*$ *is computed using a (possibly misspecified) set of cumulative distributions, denoted* $F_i(y)$ *for* Y_i.

If $G_i(y) = h\{F_i(y)\}$ *for some function* $h(\cdot)$*, then for each* i*:*

$$P_*(Y_i^* \le y) = G_i(y)$$

Proof.

$$\begin{aligned} P_*(Y_i^* \le y) = P_*\{F_i(Y^*) \le F_i(y)\} &= P_*\{U_i^* \le F_i(y)\} \\ &= \sum_{i=1}^{n} \frac{1}{n} P\{U_i \le F_i(y)\} \end{aligned}$$

since the bootstrap sample U_i^* is drawn at random with replacement from the set of observed PIT-residuals.

$$\begin{aligned} &= h\{F_i(y)\} \quad \text{from Lemma 1} \\ &= G_i(y) \end{aligned}$$

□

Lemma 2 shows that if probability integral transform residuals are identically distributed, then the Y_i^* preserve the marginal distribution of the Y_i.

Proof of Theorem 1

We will prove Theorem 1 by showing that asymptotically, the conditions of Lemma 2 are satisfied.

First note that if $\widehat{\boldsymbol{\theta}}$ is $\sqrt{n}$-consistent for $\boldsymbol{\theta}$ then provided that $F_j(y; \widehat{\boldsymbol{\theta}}, \mathbf{x}_i)$ is twice differentiable with respect to $\boldsymbol{\theta}$ then $F_j(y; \widehat{\boldsymbol{\theta}}, \mathbf{x}_i) = F_j(y; \boldsymbol{\theta}, \mathbf{x}_i) + O_p(n^{-1/2})$.

Hence, up to a term $O_p(n^{-1/2})$, $F_j(y; \widehat{\boldsymbol{\theta}}, \mathbf{x}_i)$ satisfies the conditions of Lemma 2 (where $h(\cdot)$ is the identity function). By Lemma 2, PIT-trap values follow the true cumulative distribution function $F_j(y; \boldsymbol{\theta}, \mathbf{x}_i)$, up to a term no larger than $O(n^{-1/2})$.

□

Note: While this argument uses the result that the $F_j(y; \widehat{\boldsymbol{\theta}}, \mathbf{x}_i)$ approximate the true distribution $F_j(y; \boldsymbol{\theta}, \mathbf{x}_i)$, we can relax this assumption along the lines of Lemma 1 such that there is only the requirement that the PIT-residuals are (asymptotically) identically distributed, $P(U_{ij} \leq u) = h(u)$ for each (i, j). Thus the PIT-trap can preserve the marginal distribution of the data under certain forms of model misspecification.

Proof of Theorem 3

The proof follows via the usual Edgeworth expansion approach in **?**.

If $T = g(\mathbf{Y})$ admits an Edgeworth expansion then:

$$P(T \leq t) = \Phi(t) + n^{-1/2} p_1(t)\phi(t) + n^{-1} p_2(t)\phi(t) + O(n^{-3/2})$$

where $p_1(t)$ is an odd polynomial function of the skewness of T, $p_2(t)$ is an even polynomial function of the skewness and kurtosis of T, and these moments are evaluated with respect to the distribution of the matrix of data $\mathbf{y}$, which is characterized by its margins $F(y; \boldsymbol{\theta}, \mathbf{x}_i)$, and the correlation between PIT-residuals $var(\mathbf{U}_i) = \boldsymbol{\Sigma}$.

If Y is discrete then the same type of expansion applies, but only at continuity-corrected points and not at all t (**?**).

Under the same assumptions, the distribution of the PIT-trap statistic $T^* = g(\mathbf{Y}^*)$ under resampling admits a similar Edgeworth expansion:

$$P_*(T^* \leq t) = \Phi(t) + n^{-1/2} \hat{p}_1(t)\phi(t) + n^{-1} \hat{p}_2(t)\phi(t) + O_p(n^{-3/2})$$

where $\hat{p}_1(t)$ and $\hat{p}_2(t)$ are evaluated with respect to PIT-trapped data $\mathbf{Y}^*$ whose marginal distribution is $F(y; \widehat{\boldsymbol{\theta}}, \mathbf{x}_i)$, where the correlation between PIT-trapped residuals is $var_*(\mathbf{U}_i) = \widehat{\boldsymbol{\Sigma}}$.

Now from Theorem 1, the cumulative distribution function of a PIT-trap value Y^*_{ij} is $F(y; \boldsymbol{\theta}_j, \mathbf{x}_i) + O_p(n^{-1/2})$, and from Theorem 1, $var(\mathbf{U}^*_i) = \widehat{\boldsymbol{\Sigma}}$ whose entries differ from

those of $\mathbf{\Sigma}$ by $O_p(n^{-1/2})$. Since $F(y; \boldsymbol{\theta}_j, \mathbf{x}_i)$ and $\mathbf{\Sigma}$ characterize the joint distribution of the $\mathbf{Y}_i$,

$$\hat{p}_k(t) = p_k(t) + O_p(n^{-1/2})$$

for any k for which the kth moment of Y_{ij} is defined.

Hence the coefficients of $n^{-1/2}$ in the above two Edgeworth expansions match to first order and

$$P_*(T^* \leq t) = P(T \leq t) + O_p(n^{-1})$$

As in **?**, $\hat{p}_k(t)$ and $p_k(t)$ are odd functions for odd k. Hence the odd terms cancel when calculating a two-tailed probability, removing the coefficient of $n^{-1/2}$ in each expansion, and the coefficients of n^{-1} match to first order, so

$$P_*(-t \leq T^* \leq t) = P(-t \leq T \leq t) + O_p(n^{-3/2})$$

References

Hall, P. (1992). *The bootstrap and Edgeworth expansion.* Springer-Verlag, New York.

Kolassa, J. E. and McCullagh, P. (1990). Edgeworth series for lattice distributions. *The Annals of Statistics*, 18(2):981–985.
